# Rapid Motion Segmentation of LiDAR Point Cloud Based on a Combination of Probabilistic and Evidential Approaches for Intelligent Vehicles

**DOI:** 10.3390/s19194116

**Published:** 2019-09-23

**Authors:** Kichun Jo, Sumyeong Lee, Chansoo Kim, Myoungho Sunwoo

**Affiliations:** 1 Department of Smart Vehicle Engineering, Konkuk University, Seoul 05029, Korea; 2 Department of Automotive Engineering, Hanyang University, Seoul 04763, Korea; smlee950728@gmail.com (S.L.); chansoo7857@gmail.com (C.K.); msunwoo728@gmail.com (M.S.)

**Keywords:** LiDAR, laser beam model, point motion classification, Dempster-Sharfer theory, intelligent vehicle

## Abstract

Point clouds from light detecting and ranging (LiDAR) sensors represent increasingly important information for environmental object detection and classification of automated and intelligent vehicles. Objects in the driving environment can be classified as either dynamic or static depending on their movement characteristics. A LiDAR point cloud is also segmented into dynamic and static points based on the motion properties of the measured objects. The segmented motion information of a point cloud can be useful for various functions in automated and intelligent vehicles. This paper presents a fast motion segmentation algorithm that segments a LiDAR point cloud into dynamic and static points in real-time. The segmentation algorithm classifies the motion of the latest point cloud based on the LiDAR’s laser beam characteristics and the geometrical relationship between consecutive LiDAR point clouds. To accurately and reliably estimate the motion state of each LiDAR point considering the measurement uncertainty, both probability theory and evidence theory are employed in the segmentation algorithm. The probabilistic and evidential algorithm segments the point cloud into three classes: dynamic, static, and unknown. Points are placed in the unknown class when LiDAR point cloud is not sufficient for motion segmentation. The point motion segmentation algorithm was evaluated quantitatively and qualitatively through experimental comparisons with previous motion segmentation methods.

## 1. Introduction

LiDAR systems are rapidly becoming an integral part of automated and intelligent vehicles for environmental awareness. The price of LiDAR sensors is reducing, and automakers are increasingly installing LiDAR in production vehicles for advanced intelligent functions [1,2]. LiDAR measures the distance and direction of the surrounding environment by emitting laser pulses in certain directions and measuring the time-of-flight (ToF) of each laser pulse reflected by the environment. The directions and distances can be converted to a digital 3D representation called a point cloud to express the spatial information of the surrounding environment. Because LiDAR uses light waves, the measured point cloud can represent spatial information very accurately. In addition, LiDAR point clouds can be fused with data from other sensors, such as radars, and cameras, to gain more meaningful information about the vehicle’s environment.

All objects in the driving environment are classified as dynamic or static according to the moving conditions. Therefore, the LiDAR point cloud from detected objects can also be classified as dynamic or static point-wise depending on the motion state of the object. Such point-wise classification of point cloud states can be used for safety and convenience functions in automated and intelligent vehicles. For instance, points classified as static are measured from the surfaces of static objects, such as curbs, poles, buildings, and parked vehicles. Such a static point cloud can be applied to various automated and intelligent driving functions, such as mapping, localization, and collision avoidance systems [3,4,5]. Points classified as dynamic are detected from objects that have speeds above a certain level, such as nearby moving vehicles, motorcycles, and pedestrians. These points can be used for object tracking or motion prediction, which are necessary functions for automated and intelligent vehicles for tasks such as autonomous emergency braking (AEB), lane keeping, traffic jam assistance, and adaptive cruise control (ACC) systems.

**Figure 1:**
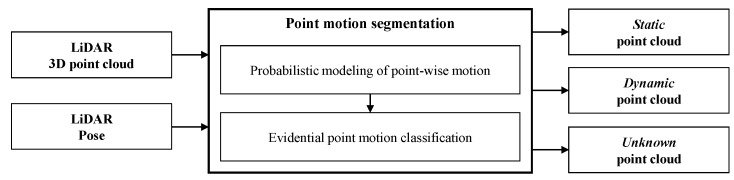
System architecture of the rapid point motion segmentation algorithm.

As shown in the previous examples, the information of the point cloud state is classified according to the motion is useful for automated and intelligent vehicles. This paper proposes an algorithm to rapidly segment the motion states of a point cloud detected by LiDAR in real-time. The overall process of the proposed algorithm is shown in Figure 1. The rapid motion segmentation algorithm has inputs of LiDAR’s 3D point cloud and the 3D pose (position and direction) of the LiDAR sensor. The sensor pose can be estimated from an inertial measurement unit (IMU) or the vehicle’s on-board motion sensors (such as wheel speed sensors or steering angle sensor). Then, point motion segmentation is performed by applying the laser beam characteristics to the pose correlation between consecutive LiDAR point clouds. A combination of probability theory and evidence theory is applied to accurately and reliably update the motion state of points. The algorithm performs point-wise segmentation of the point cloud into three states: dynamic, static, and unknown. dynamic information is detected from an object moving above a certain speed, and static information is detected from a stationary object. If there are insufficient consecutive LiDAR point clouds for motion classification, some points are classified as unknown. The performance of the proposed algorithm was evaluated quantitatively and qualitatively through comparison with existing methods.

This research has three main contributions: (1) reflecting the laser characteristics of LiDAR, (2) applying a combination of probabilistic and evidential approaches to update the motion state of points, and (3) online motion updated for real-time applications. This paper focuses on the characteristics of lasers, such as multi-echo, beam divergence, and horizontal and vertical resolution, so that it can segment the motion of points more accurately than existing algorithms, such as occupancy grid mapping. In addition, when updating the state information, a combination of probabilistic and evidential modeling is applied to more accurately reflect the actual LiDAR characteristics to update motion in a point-wise manner. Because all the proposed updating processes are real-time, they are suitable for real-time application in automated and intelligent vehicle systems.

## 2. Previous Studies

With the development of autonomous vehicles, LiDAR is being more widely used, and many studies of LiDAR are accordingly being conducted. However, there have not been many studies that classify the point-wise motion of LiDAR measurements themselves in real-time. In the field of autonomous and intelligent vehicle systems, studies related to the proposed algorithm aim to generate static environment maps or remove nonstatic points based on tracking results.

An occupancy grid map is a typical method for generating a static environment map using LiDAR measurements. The occupancy grid map divides the environment around the vehicle into a 2D grid or 3D voxel cells with uniform size. The occupancy level of each cell can be updated through the laser’s ray tracing. The occupancy level of a grid (or voxel) cell passing by the laser becomes lower because the physical space of the cell is likely to be free. Conversely, the levels of cells located on the reflecting surface become higher. Based on these principles, static objects are classified as occupied when the occupancy level exceeds a certain threshold. Contrarily, for a moving object, the occupancy level of the cell is not constantly accumulated, so it is not classified as occupied. The numerical value of each cell’s occupancy level is updated based on probability theory [6] or belief theory [7]. Static maps in large-scale traffic environment are constructed using LiDAR sensors with probabilistic and belief approaches [8,9,10]. Moras et al. presented an occupancy grid framework that generates a global static map and classifies local moving objects simultaneously [11,12,13]. Classification of traffic objects (such as vehicles, pedestrians, road curbs, and poles) is used to classify the motion of a point cloud [14,15,16].

The advantages of occupancy grid-based static point cloud classification are that its implementation is relatively straightforward and its performance is stable because it has been studied for a long time in various applications. However, occupancy grid mapping has several disadvantages for use in real-time automated and intelligent vehicle applications. Large memory is required because the driving environment must be represented by a grid or voxel cells. Also, the ray tracing method takes a long time to update all cells related to all LiDAR beams. In addition, because space is represented by discrete cells, a discretization error occurs when the resolution is coarse.

Research on the detection and tracking of moving objects using LiDAR has been conducted to recognize the driving environment of automated and intelligent vehicles [17,18]. Object detection algorithms detect surrounding objects by clustering the point cloud and generate a bounding box for the each detected object. Tracking algorithms generate tracks for detected objects to estimate their position, direction, velocity, and acceleration. Using the tracking results, we can classify the motion of a point cloud into dynamic and static states. Points in a track bounding box above a certain speed are classified as dynamic, and the remaining points are classified as static. This tracking-based point motion classification is straightforward, and the tracking results can be reused. However, it has some limitations. The point cloud clustering groups the detected points on the same object in the object detection step. Although many clustering methods have been studied, it is difficult to obtain accurate results using point cloud information alone. Incorrect clustering causes incorrect point motion classification. In addition, because the tracking has an initialization time to generate new track, it struggles to satisfy real-time motion classification. Furthermore, because it is difficult for tracking to accurately estimate the speed of slow objects, the point motion of objects is likely to be misclassified near the threshold speed.

The point motion classification algorithm presented in this paper has many advantages over previously proposed ones. First, the proposed method directly segments the point cloud into dynamic and static states using the laser beam model. Therefore, there is no chance of misclassification due to discretization error in the occupancy grid method and erroneous clustering of the tracking-based method. In addition, the proposed method does not require a large amount of memory like the occupied grid approach because it simply buffers recent point clouds. Finally, the proposed method is able to satisfy the real-time requirements of point motion classification because it does not need to update all of the gird cells to initialize a new track.

## 3. System Architecture

The objective of the proposed point motion segmentation algorithm is to classify the latest LiDAR point, $z_{t}^{n,m}$, into motion states, $motion_{t}^{n,m}$, in real-time. The notation of the LiDAR point and motion states are $z_{time}^{index,order}$ and $motion_{time}^{index,order}$, respectively. The *n* describes the index of the laser beam from 1 to *N*. The *m* represents the order of multi-echo for the laser beam and usually has a value of 2 or less. The *t* represents the time for the LiDAR scan measurement. The “motion state” has three possible values: motion={dynamic,static,unknown}. The dynamic state indicates the points detected as moving objects, and the static state represents points detected as stationary objects. The unknown state means that there is not sufficient evidence to classify the motion state as dynamic or static.

The basic principle of the proposed algorithm is to classify the motion state of the current point cloud by applying the laser characteristic model to the registration relationship between the previously measured point cloud and the current point cloud. The inputs of the proposed algorithm are the current point cloud, $Z_{t}=\left\{z_{t}^{1,m},z_{t}^{2,m},\cdots ,z_{t}^{N,m}\right\}$; the previously buffered point clouds, $Z_{t-1},\cdots ,Z_{t-W+1},Z_{t-W}$; and the sensor pose, $x_{t},x_{t-1},\cdots ,x_{t-W+1},x_{t-W}$, for each point cloud. *W* denotes the time window size of the previous data buffer to be used for motion classification of the current point cloud. There are several methods for obtaining the sensor pose $x_{t},\cdots ,x_{t-W}$, such as inertial measurement unit (IMU) dead reckoning, scan matching, a high-definition (HD) map-based localization, and simultaneous localization and mapping (SLAM). To avoid loss of generality, we assume that the sensor’s pose and its uncertainty are provided. The output of the algorithm is the motion state $Motion_{t}=\left\{motion_{t}^{1,m},motion_{t}^{2,m},\cdots ,motion_{t}^{N,m}\right\}$ of $Z_{t}=\left\{z_{t}^{1,m},z_{t}^{2,m},\cdots ,z_{t}^{N,m}\right\}$.

**Figure 2:**
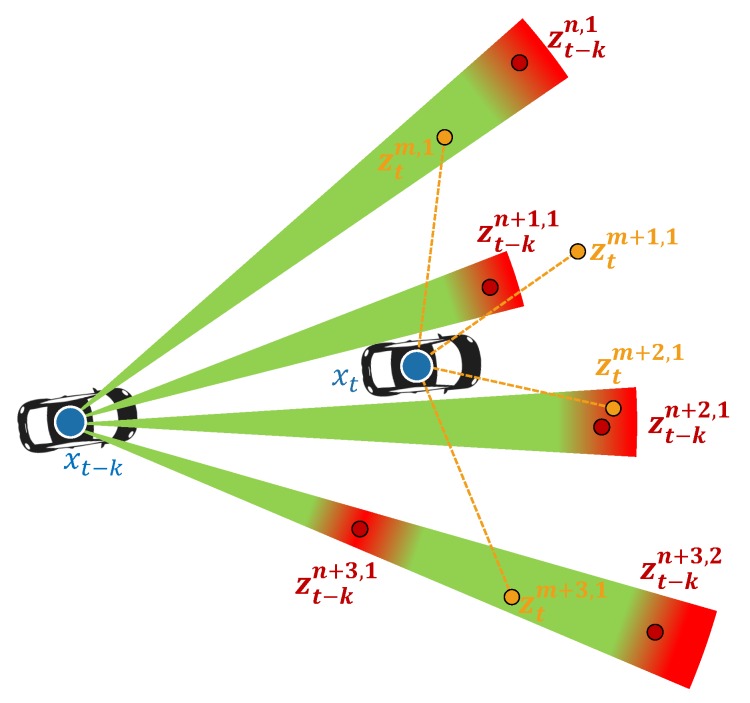
Probability update of $Motion_{(t-k)\to t}$. The motion probability of the point cloud $Z_{t}$ can be updated based on the geometric relationship for the previous point cloud $Z_{t-k}$.

The point motion segmentation algorithm consists of two steps: (1) probabilistic modeling of point motion and (2) evidential point motion classification. In the first step, the probability of $Motion_{(t-k)\to t}$, which is a motion classification of $Z_{t}$ against $Z_{t-k}$, is updated. Figure 2 illustrates the concept of the probability update of $Motion_{(t-k)\to t}$ based on a geometrical relationship between $\left(Z_{t},x_{t}\right)$ and $\left(Z_{t-k},x_{t-k}\right)$. The likelihood field of the motion can be updated using $\left(Z_{t-k},x_{t-k}\right)$ and the characteristics of the laser (such as beam divergence and multi-echo). The Dynamic probability for $z_{t}^{m,1}$ and $z_{t}^{m+3,1}$ will be higher when they are located in the path of the laser (green region) for the previous point cloud $Z_{t-k}$, and the Static probability for $z_{t}^{m+2,1}$ will be higher if it is located near the previous point (red region).

**Figure 3:**
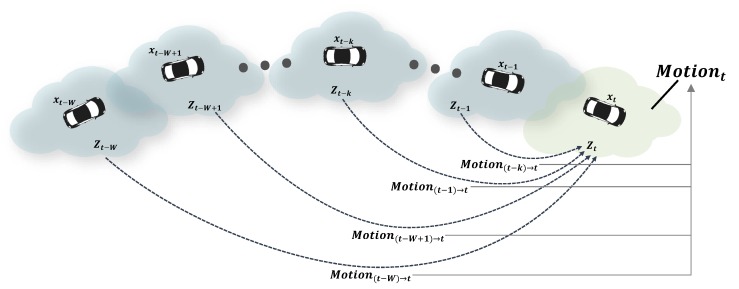
Motion probabilities $p\left(Motion_{(t-1)\to t}\right),\cdots ,p\left(Motion_{(t-W)\to t}\right)$ are integrated into the latest motion probability $p(Motion_{t})$.

In the second step, the probabilities of each motion classification $Motion_{(t-1)\to t},\cdots ,Motion_{(t-W)\to t}$ are integrated to estimate the final motion classification $Motion_{t}$, as shown in Figure 3. However, the probability of $Motion_{(t-k)\to t}$ cannot be updated by previous points if the current points are not in the likelihood field of the previous points. In this case, it should be classified as unknown. However, because unknown cannot be expressed clearly using probability theory, evidence theory, which can handle the unknown state explicitly, is employed. The probabilities of $Motion_{(t-1)\to t},\cdots ,Motion_{(t-W)\to t}$ are converted into mass (degree of belief) with consideration of LiDAR and sensor pose uncertainty and then integrated into a mass of $Motion_{t}$ using Dempster’s combination rule. The motion states $Motion_{t}=\left\{motion_{t}^{1,m},motion_{t}^{2,m},\cdots ,motion_{t}^{N,m}\right\}$ of each point $Z_{t}=\left\{z_{t}^{1,m},z_{t}^{2,m},\cdots ,z_{t}^{N,m}\right\}$ are determined using the integrated mass of $Motion_{t}$.

## 4. Probabilistic Modeling of LiDAR Point Motion

### 4.1. Characteristics of LiDAR Point Cloud

**Figure 4:**
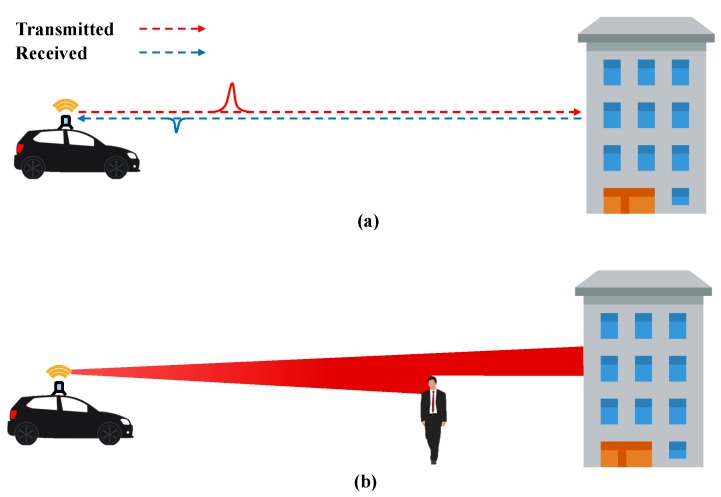
Light detection and ranging (LiDAR) measurement characteristics: (**a**) Time-of-flight (ToF) and (**b**) laser beam divergence and multi-echo.

LiDAR uses rotating laser beams to measure the distances and angles from surrounding objects. A laser pulse is emitted at a specific angle, and the distance to the object for that angle can be measured using the time-of-flight (ToF) principle, as demonstrated in Figure 4. ToF represents the difference between the time the laser pulse is emitted from the diode and the time it returns to the object. The distance is calculated by multiplying this time by the speed of the laser light. Using the horizontal–vertical emitted angles and the measured distances, 3D information of surrounding objects can be reconstructed in the form of point data.

The actual LiDAR’s laser is not emitted in a straight line, as shown in the Figure 4a. The laser has a characteristic of “beam divergence”, which increases the beam’s cross section over the distance. Because of this characteristic, the farther away from the laser source an object is, the wider the area objects can be detected in. In addition, beam divergence enables multi-echoing of the emitted laser pulse, as shown in Figure 4b, and the multi-echo allows simultaneous measurement of distances to various objects. Another important characteristic of LiDAR is the uncertainty of the distance and angular measurement. Despite the use of lasers, the distance measurement is not infinitely accurate. The distance measurement accuracy is proportional to the measuring capability of the time the laser pulse takes to return. Conversely, the angular accuracy (vertical and horizontal) is discretely accurate because LiDAR is able to control and configure the emission angle. Many previous studies that used LiDAR measurements did not properly account for the above-mentioned characteristics of LiDAR (i.e., beam divergence and distance uncertainty); they treated LiDAR measurements as points with no volume and constant 3D Gaussian uncertainty. To classify LiDAR point motion with high accuracy and reliability, the proposed algorithm accurately reflects these characteristics of LiDAR.

The LiDAR point cloud measurement $Z_{t}=\left\{z_{t}^{1,m},z_{t}^{2,m},\cdots ,z_{t}^{N,m}\right\}$ must be representable in both spherical and Cartesian coordinates for processing by the point motion segmentation algorithm. The measurement can be represented in spherical coordinates as $Z_{r\theta \phi ,t}=\left\{z_{r\theta \phi ,t}^{1,m},\cdots ,z_{r\theta \phi ,t}^{i,m},\cdots ,z_{r\theta \phi ,t}^{N,m}\right\}$. The point $z_{r\theta \phi ,t}^{i,m}$ is represented as $z_{r\theta \phi ,t}^{i,m}=\left\{r_{t}^{i,1},\cdots ,r_{t}^{i,m},\theta_{t}^{i},\phi_{t}^{i}\right\}$, where *r* is distance to a point with a second echo, θ is the vertical (polar) angle of the point, ϕ is the horizontal (azimuthal) angle of the point, and *m* is the number of echos. The Cartesian coordinate representation is $Z_{xyz,t}=\left\{z_{xyz,t}^{1,m},\cdots ,z_{xyz,t}^{i,m},\cdots ,z_{xyz,t}^{N,m}\right\}$, where $z_{xyz,t}^{i,m}=\left\{x_{t}^{i,m},y_{t}^{i,m},z_{t}^{i,m}\right\}$.

### 4.2. Probabilistic Modeling for LiDAR Point Motion

The point motion segmentation classifies the latest *N* LiDAR points $z_{t}^{1,m},z_{t}^{2,m},\cdots ,z_{t}^{N,m}$ into the motion states $motion_{t}^{1,m},motion_{t}^{2,m},\cdots ,motion_{t}^{N,m}$, respectively, in real-time, where motion consists of three states {dynamic,static,unknown}. The motion state of the latest point cloud is segmented based on the registration relationship for the previously buffered point cloud. In other words, the latest point cloud $Z_{t}=\left\{z_{t}^{1,m},z_{t}^{2,m},\cdots ,z_{t}^{N,m}\right\}$ is segmented into $Motion_{t}=\left\{motion_{t}^{1,m},motion_{t}^{2,m},\cdots ,motion_{t}^{N,m}\right\}$ based on the *W*-buffered point clouds $Z_{t-1},\cdots ,Z_{t-W+1},Z_{t-W}$ and the sensor pose $x_{t},x_{t-1},\cdots ,x_{t-W+1},x_{t-W}$ of each point cloud. The sensor pose $x_{t},\cdots ,x_{t-W}$ can be obtained using several methods, such as an IMU dead reckoning, scan matching, HD map-based localization, and SLAM. However, the proposed algorithm assumes that the sensor’s pose information and its uncertainty is abstracted regardless of the type of pose estimation method.

Probabilistic motion modeling of the point cloud $Z_{t}$ can be estimated using the LiDAR sensor pose $x_{t}$ of $Z_{t}$ and the previously detected LiDAR point cloud $Z_{t-k}$ and its sensor pose $x_{t-k}$, as shown in Figure 2. The probabilistic motion model of $Z_{t}$ to $x_{t}$, $Z_{t-k}$, and $x_{t-k}$ can be described as $p(Motion_{(t-k)\to t})$. The probability $p(Motion_{(t-k)\to t})$ can be represented by a conditional probability for the given conditions, the past and present point cloud pairs $\left(Z_{t},Z_{t-k}\right)$, and their sensor pose $\left(x_{t},x_{t-k}\right)$, as represented in Equation (1).

(1) $$p\left(Motion_{(t-k)\to t}\right)=p\left(Motion_{t}|Z_{t},Z_{t-k},x_{t},x_{t-k}\right)$$

$Motion_{t}$ is composed of each independent point motion $\left\{motion_{t}^{1,m},motion_{t}^{2,m},\cdots ,motion_{t}^{N,m}\right\}$, so $p(Motion_{(t-k)\to t})$ can be represented by the set of conditional probabilities of each point, as described by

(2) $$p\left(Motion_{(t-k)\to t}\right)=\left\{p\left(motion_{t}^{1,m}|Z_{t},Z_{t-k},x_{t},x_{t-k}\right),\cdots ,p\left(motion_{t}^{N,m}|Z_{t},Z_{t-k},x_{t},x_{t-k}\right)\right\}.$$

$motion_{t}^{i,m}$ consists of two states {dynamic,static}, and the sum of p(dynamic) and p(static) is always one.

The conditional probability of one point motion can be reorganized by the Bayes rule, as represented by Equation (3).

(3) $$p\left(motion_{t}^{i,m}|z_{t}^{i,m},Z_{t-k},x_{t},x_{t-k}\right)=\frac{p\left(z_{t}^{i,m}|motion_{t}^{i,m},Z_{t-k},x_{t},x_{t-k}\right)p\left(motion_{t}^{i,m}|Z_{t-k},x_{t},x_{t-k}\right)}{p(z_{t}^{i,m}|Z_{t-k},x_{t},x_{t-k})}$$

$p(z_{t}^{i,m}|motion_{t}^{i,m},Z_{t-k},x_{t},x_{t-k})$ is the likelihood of the LiDAR point measurement for the given motion state. $p(motion_{t}^{i,m}|Z_{t-k},x_{t},x_{t-k})$ is the predicted probability density function. The motion for given $Z_{t-k}$, $x_{t}$, and $x_{t-k}$ can be represented by a uniform distribution, so $p(motion_{t}^{i,m}=\left\{\mathrm{static}\;\mathrm{or}\;\mathrm{dynamic}\right\}|Z_{t-k},x_{t},x_{t-k})$ is 0.5. $p(z_{t}^{i,m}|Z_{t-k},x_{t},x_{t-k})$ can be a normalization factor by applying marginalization, as described by Equation (4).

(4) $$p\left(motion_{t}^{i,m}|z_{t}^{i,m},Z_{t-k},x_{t},x_{t-k}\right)=\sum_{motion}p\left(z_{t}^{i,m}|motion_{t}^{i,m},Z_{t-k},x_{t},x_{t-k}\right)p\left(motion_{t}^{i,m}|Z_{t-k},x_{t},x_{t-k}\right)$$

By summarizing the above equations, the posterior probability of one point motion can be represented by the following equation,

(5) $$p\left(motion_{t}^{i,m}|z_{t}^{i,m},Z_{t-k},x_{t},x_{t-k}\right)=\eta \;p\left(z_{t}^{i,m}|motion_{t}^{i,m},Z_{t-k},x_{t},x_{t-k}\right)$$

where η is the normalization factor $p(motion_{t}^{i,m}|z_{t}^{i,m},Z_{t-k},x_{t},x_{t-k})$ of Equation (4). Equation (5) is the conditional probability of one LiDAR point motion being expressed by the likelihood of the given point motion. Therefore, the motion probability estimation problem is converted to a likelihood estimation problem for LiDAR point cloud.

### 4.3. Likelihood of LiDAR Point Measurement

**Figure 5:**
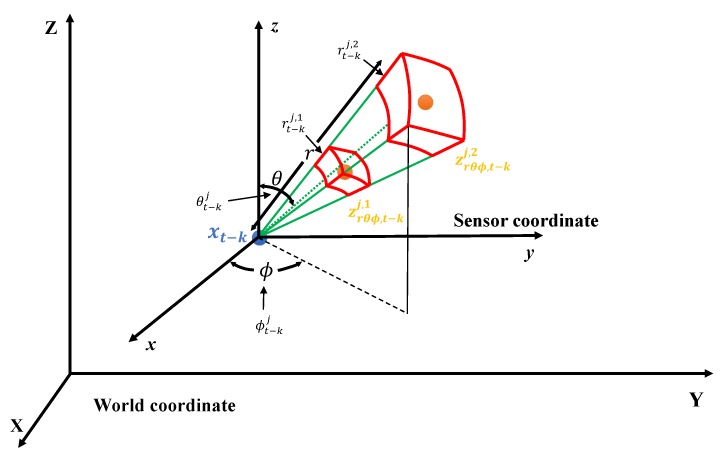
Likelihood field for the point $z_{t}^{i,m}$ is constructed by previous point $z_{r\theta \phi ,t-k}^{j,l}$ with consideration of the beam divergence.

We know that the point cloud motion probability $p(Motion_{(t-k)\to t})$ can be obtained from the likelihood of the point cloud $p(z_{t}^{i,m}|motion_{t}^{i,m},Z_{t-k},x_{t},x_{t-k})$, as described by Equation (5). The likelihood $p(z_{t}^{i,m}|motion_{t}^{i,m},Z_{t-k},x_{t},x_{t-k})$ represents a statistical state when $motion_{t}^{i,m}$ is determined as static or dynamic for given $Z_{t-k}$, $x_{t}$, and $x_{t-k}$. The likelihood field of one point $z_{t}^{i,m}$ can be represented intuitively, as shown in Figure 5. The likelihood field of the LiDAR point $z_{t}^{i,m}$, measured at sensor pose $x_{t}$ at time *t*, is represented by points $z_{t-k}^{j,l}$ in point cloud $Z_{t-k}$ measured at pose $x_{t-k}$ at the previous time t−k. The intensity of the green color indicates the likelihood of the point $z_{t}^{i,m}$ in the green region being in dynamic motion, and the intensity of the red region represents the likelihood of the point $z_{t}^{i,m}$ in the red region being in static motion. The likelihood field is represented in the local spherical coordinates of the previous sensor pose $x_{t-k}$. The point cloud $Z_{t-k}$ is represented in spherical coordinates as $Z_{t-k}=Z_{r\theta \phi ,t-k}=\left\{z_{r\theta \phi ,t-k}^{1,l},\cdots ,z_{r\theta \phi ,t-k}^{j,l},\cdots ,z_{r\theta \phi ,t-k}^{J,l}\right\}$. The likelihood field for the point $z_{t}^{i,m}$ is constructed in a triangular-pyramid form by each previous point $z_{r\theta \phi ,t-k}^{j,l}$ with consideration of the beam divergence characteristics of the LiDAR laser, as shown in Figure 5.

**Figure 6:**
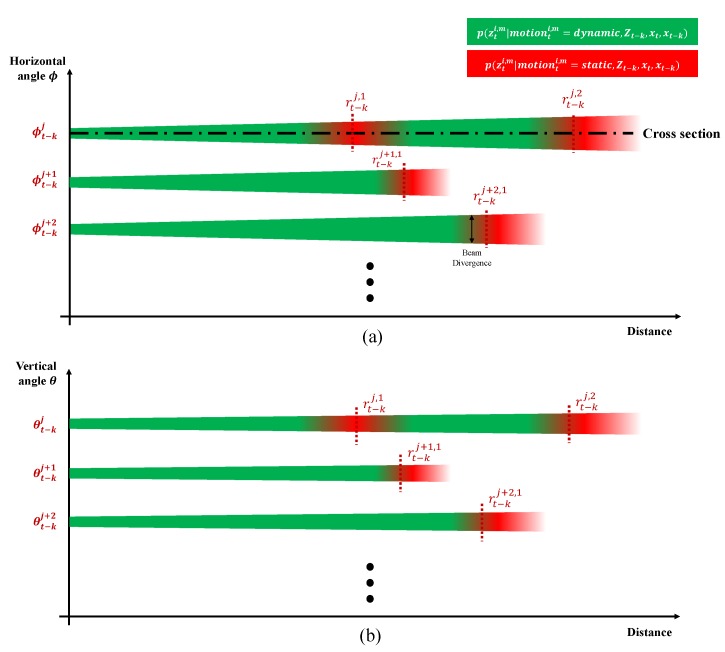
2D likelihood field for the distance (*r*) and angle (horizontal ϕ (**a**) and vertical θ (**b**)) of the previous measurements $z_{t-k}^{j,1},z_{t-k}^{j,2},z_{t-k}^{j+1,1}$ and $z_{t-k}^{j+2,1}$.

The 3D likelihood distribution of $p(z_{t}^{i,m}|motion_{t}^{i,m},Z_{t-k},x_{t},x_{t-k})$ can be divided by two 2D likelihood fields. The first one is a likelihood field for the distance–horizontal angle (r−ϕ) plane, $p(r_{t}^{i,m},\phi_{t}^{i}|motion_{t}^{i,m},Z_{t-k},x_{t},x_{t-k})$, and the second is a likelihood field of the distance-vertical angle (r−θ) plane, $p(r_{t}^{i,m},\theta_{t}^{i,m}|motion_{t}^{i},Z_{t-k},x_{t},x_{t-k})$. Figure 6 shows the 2D likelihood field in the (r−ϕ) and (r−θ) planes for the previous measurements $z_{t-k}^{j,1},z_{t-k}^{j,2},z_{t-k}^{j+1,1}$ and $z_{t-k}^{j+2,1}$. For each horizontal and vertical angle, the likelihood field is distributed discretely based on the resolution of the horizontal and vertical laser pulses. Due to the beam divergence of the laser, the likelihood fields gradually disperse.

**Figure 7:**
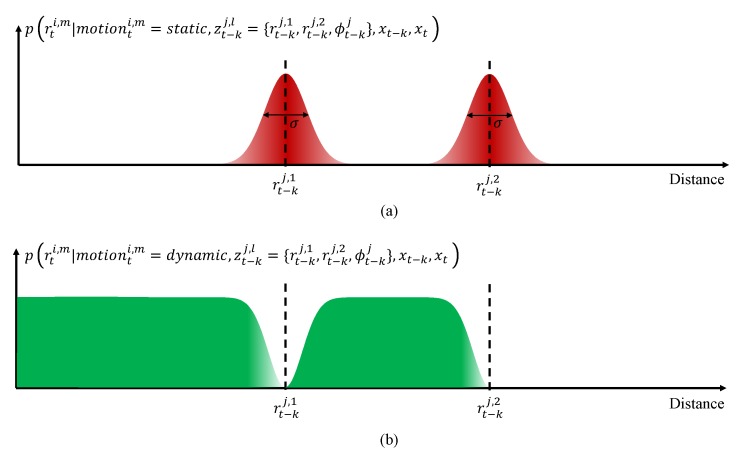
Likelihood of static (**a**) and dynamic (**b**) for one laser beam.

The cross-section of the likelihood field for one laser beam $z_{t-k}^{j,l}$ in Figure 6a can be represented by the likelihood value in Figure 7. Through this figure, we can more accurately analyze the distribution of likelihood for each motion. Figure 7a shows the likelihood $p(z_{t}^{i,m}|motion_{t}^{i,m}=static,z_{t-k}^{j,l},x_{t},x_{t-k})$ when a measured point $z_{t}^{i,m}$ is static for given previous measurement $z_{t-k}^{j,l}$ and given poses $x_{t}$ and $x_{t-k}$. Here, the previous measurement $z_{t-k}^{j,l}$ can be expressed in spherical coordinates as $\left\{r_{t-k}^{j,1},r_{t-k}^{j,2},\phi_{t-k}^{j}\right\}$. The region where the previous LiDAR point was detected is likely to be static. LiDAR is measured using ToF, so the uncertainty of the measured distance, $r_{t}^{i,m}$, depends on the accuracy of the ToF sensor. Considering this uncertainty, the likelihood of static motion can be expressed as a Gaussian distribution, as described by Figure 7a and Equation (6).

(6) $$p\left(r_{t}^{i,m}|motion_{t}^{i,m}=static,z_{t-k}^{j,l},x_{t},x_{t-k}\right)=\frac{1}{\sigma \sqrt{2\pi }}e^{-{\left(r-r_{t}^{i,m}\right)}^{2}/2\sigma^{2}}$$

σ is the standard deviation of the distance measurement, which are different depending on the LiDAR. Figure 7b shows the likelihood $p(z_{t}^{i,m}|motion_{t}^{i,m}=dynamic,z_{t-k}^{j,l},x_{t},x_{t-k})$ when a measured point $z_{t}^{i,m}$ is dynamic for given $z_{t-k}^{j,l},x_{t}$, and $x_{t-k}$. The region where the previous LiDAR beam $z_{t-k}^{j,l}$ passed is likely to be free, and the location of the current LiDAR point $z_{t}^{i,m}$ in the region means that this point is more likely to be detected from a dynamic object. This characteristic can be represented by the following equation:

(7) $$\begin{array}{ll} p(r_{t}^{i,m}|motion_{t}^{i,m} & =dynamic,z_{t-k}^{j,l},x_{t},x_{t-k})=\\ & \left\{\begin{array}{cc} MaxLikelihood_{t-k}^{i,m}-p\left(r_{t}^{i,m}|motion_{t}^{i,m}=static,z_{t-k}^{j,l},x_{t},x_{t-k}\right) & r<=r_{t-k}^{j,max(m)}\\ 0 & else \end{array}\right. \end{array}$$

Here, $MaxLikelihood_{t-k}^{i,m}$ denotes the maximum likelihood value for the previous point measurement $z_{t-k}^{j,l}$ and can be represented by the following equation:

(8) $$MaxLikelihood_{t-k}^{i,m}=p\left(r_{t-k}^{j,l}|motion_{t}^{i,m}=static,z_{t-k}^{j,l},x_{t},x_{t-k}\right)$$

When the point $z_{t}^{i,m}$ is located in the likelihood field for the given $\left\{z_{t-k}^{j,l},x_{t-k},x_{t}\right\}$, we can obtain the likelihood through Equations (6) and (7). Then, the probability of the point motion can be calculated through Equation (5). However, if the point $z_{t}^{i,m}$ is located outside of the likelihood field, we cannot obtain the likelihood using the above equations. The area outside of the likelihood field must be dealt with as unknown, but probability theory cannot handle the unknown state explicitly. Therefore, in the next chapter, we apply evidence theory to deal with the unknown state explicitly.

## 5. Evidential Point Motion Classification

### 5.1. Evidential Modeling of LiDAR Point Motion

**Figure 8:**
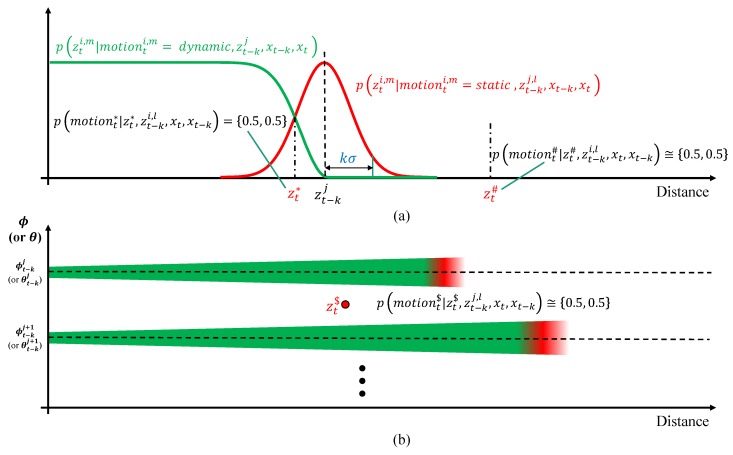
Problems of probabilistic motion segmentation. The probabilities of motion for $z_{t}^{\#}$ (**a**) and $z_{t}^{\$}$ (**b**) are close to {0.5,0.5}, because both likelihoods for dynamic and static are zero.

The process of point-wise probabilistic motion estimation achieved through the likelihood field was described in the previous section. However, there is a limitation to the probabilistic method if the point $z_{t}^{i,m}$ is not located above the likelihood field. We can see this limitation in Figure 8. In Figure 8a, the measurement $z_{t}^{*}$ represents a normal case because the point is located inside the likelihood field. The point motion probability $p(motion_{t}^{*}|z_{t}^{*},z_{t-k}^{i},x_{t},x_{t-k})$ can be {static,dynamic}={0.5,0.5} because the likelihoods for dynamic and static are the same. However, the probabilities of motion for $z_{t}^{\#}$ in Figure 8a and $z_{t}^{\$}$ in Figure 8b are not included in the likelihood field close to {0.5,0.5}, because both likelihoods for dynamic and static are zero. This means that we cannot distinguish the difference between $z_{t}^{*}$, $z_{t}^{\#}$, and $z_{t}^{\$}$ in the probabilistic approach. To overcome this limitation, an evidential approach (Dempster–Shafer theory) is applied to explicitly distinguish the motion of points that are not located in the likelihood field.

Both probabilistic and evidential approaches are based on the concept of assigning weights to the hypothesized states of the measurement. However, the evidential approach allows sets of alternatives, which means new states can be created by combining existing states. The probabilistic approach deals with the two states {static,dynamic}. In the evidential approach, the two states form a frame of discernment Ω={static,dynamic}. Dempster–Shafer theory can manage more states explicitly (Ω, ϕ) by extending the frame of discernment Ω to the power set $2^{\Omega }=\left\{static,dynamic,\Omega ,\phi \right\}$. Ω is the set Ω={static,dynamic}, which means that the point motion is static or dynamic. However, because the point motion cannot be static and dynamic simultaneously, the state Ω indicates an unknown state. ϕ is an empty set, which means that the point motion is not both static and dynamic. However, because this situation is physically impossible, the state ϕ indicates a conflict situation. For each state of the power set $2^{\Omega }=\left\{static,dynamic,unknown,conflict\right\}$ in the evidential approach, a mass function Mass is used to quantify the belief of the hypothesis. The mass functions of $Mass_{t}^{i,m}\left(static\right)$ and $Mass_{t}^{i,m}\left(dynamic\right)$ represent the belief of point $z_{t}^{i,m}$ being static and dynamic, respectively. The mass function of $Mass_{t}^{i,m}\left(unknown\right)$ is the union of the beliefs of static and dynamic, and $Mass_{t}^{i,m}\left(conflict\right)$ represents the belief that the point is conflicted by different measurements. The sum of mass functions for the power set must be one based on its definition in the evidential framework.

Based on the evidential approach, we can explicitly handle the points located outside the likelihood field for the given point $z_{t-k}^{j,l}$ as an unknown state. The boundary between the inside and outside is kσ, as shown in Figure 8. *k* is the tuning factor, which determines the size of the likelihood boundary, and we used k=3. The point $z_{t}^{*}$ is located inside the likelihood field, but $z_{t}^{\#}$ and $z_{t}^{\$}$ are located outside the likelihood field. The mass of point $z_{t}^{i,m}$ motion for the given $z_{t-k}^{j,l},x_{t}$, and $x_{t-k}$ is denoted by $mass_{(t-k)\to t}^{j,l\to i,m}\left(state\right)$ for each state={static,dynamic,unknown,conflict}. $mass_{(t-k)\to t}^{j,l\to i,m}\left(state\right)$ can be calculated based on whether the point $z_{t}^{i,m}$ is located inside or outside of the likelihood field using the following equation.

(9) $$\begin{array}{cc} mass_{(t-k)\to t}^{j,l\to i,m}\left(static\right) & =\left\{\begin{array}{cc} \lambda_{k}p\left(motion_{t}^{i,m}=static|z_{t}^{i,m},z_{t-k}^{j,l},x_{t},x_{t-k}\right), & \mathrm{inside}\\ 0, & \mathrm{outside} \end{array}\right.\\ mass_{(t-k)\to t}^{j,l\to i,m}\left(dynamic\right) & =\left\{\begin{array}{cc} \lambda_{k}p\left(motion_{t}^{i,m}=dynamic|z_{t}^{i,m},z_{t-k}^{j,l},x_{t},x_{t-k}\right), & \mathrm{inside}\\ 0, & \mathrm{outside} \end{array}\right.\\ mass_{(t-k)\to t}^{j,l\to i,m}\left(unknown\right) & =\left\{\begin{array}{cc} 1-Mass_{(t-k)\to t}^{j,l\to i,m}\left(static\right)-Mass_{(t-k)\to t}^{j,l\to i,m}\left(dynamic\right), & \mathrm{inside}\\ 1, & \mathrm{outside} \end{array}\right.\\ mass_{(t-k)\to t}^{j,l\to i,m}\left(conflict\right) & =0 \end{array}$$

The mass values of the static and dynamic states are calculated by the motion probability $p(motion_{t}^{i,m}|z_{t}^{i,m},z_{t-k}^{j,l},x_{t},x_{t-k})$ and its confidence, $\lambda_{k}$. The confidence $\lambda_{k}$ can be determined using Equation (10).

(10) $$\lambda_{k}=\lambda_{reg}exp\left(-\frac{k}{\tau }\right)$$

$\lambda_{reg}$ describes the confidence of the pose registration between $x_{t}$ and $x_{t-1}$. This value is determined by the performance of the registration method, such as IMU, scan matching, and HD mapping. If the registration is very accurate, the value is close to one; however, if it is not good, it is close to zero. The confidence $\lambda_{k}$ is also affected by the time difference *k*. Because the confidence of the probabilistic model decreases as the time difference *k* increases, the confidence $\lambda_{k}$ also decreases by exp(−k/τ), where τ is the time constant that determines the decay rate.

### 5.2. Point Motion Segmentation by Integrating the Point Motion Masses

For the given point $z_{t-k}^{j,l}$ and the given poses $x_{t}$ and $x_{t-k}$, the point motion of $z_{t}^{i,m}$ can be described by mass function $mass_{(t-k)\to t}^{j,l\to i,m}\left(state\right)$. For all given previous scan points $Z_{t-k}=\left\{z_{t-k}^{1,l},\cdots ,z_{t-k}^{j,l},\cdots ,z_{t-k}^{N,l}\right\}$ and the given poses $x_{t}$ and $x_{t-k}$, several mass functions $mass_{(t-k)\to t}^{1,l\to i,m}\left(state\right)\cdots mass_{(t-k)\to t}^{N,l\to i,m}\left(state\right)$ can be calculated. We must integrate the mass functions into one mass function $mass_{(t-k)\to t}^{i,m}\left(state\right)$. In addition, for the previously buffered point clouds $Z_{t-1},\cdots ,Z_{t-W+1},Z_{t-W}$ in the time window *W*, several mass functions $Mass_{(t-1)\to t}^{i,m}\left(state\right),\cdots ,Mass_{(t-W)\to t}^{i,m}\left(state\right)$ can be obtained, and these mass functions should be integrated into a single mass function $Mass_{t}^{i,m}\left(state\right)$ to represent the motion of one point $z_{t}^{i,m}$. To integrate two different mass values from different laser scans and times, Dempster’s combination rule (Equation (11)) is applied.

(11) $$\begin{array}{c} Mass_{1}⊕Mass_{2}⟹\\ Mass_{1⊕2}\left(A\right)=\frac{Mass_{1\cap 2}\left(A\right)}{1-Mass_{1\cap 2}\left(\phi \right)},Mass_{1⊕2}\left(\phi \right)=0, \end{array}$$

(12) $$\begin{array}{c} \forall A\subseteq \Omega ,A\neq \phi \end{array}$$

Dempster’s combination rule is based on the conjunctive combination rule described by Equation (13).

(13) $$Mass_{1\cap 2}\left(A\right)=\sum_{B\cap C=A|B,C\subseteq \Omega }Mass_{1}\left(B\right)\;\cdot \;Mass_{2}\left(B\right)$$

## 6. Experiments

### 6.1. Experimental Environments

**Figure 9:**
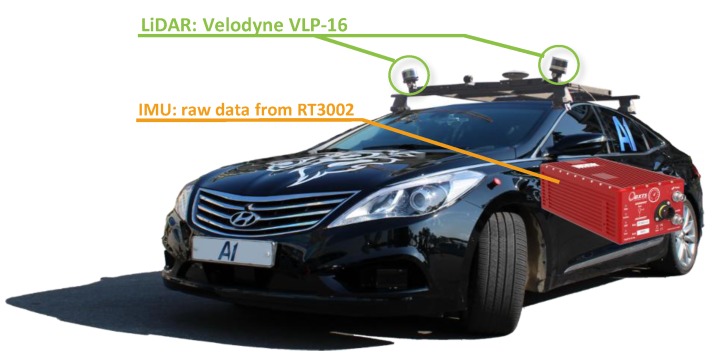
Test vehicle and sensor (LiDAR and positioning system) configuration.

An autonomous vehicle (A1) was used for the experiment to evaluate the proposed algorithm. A1 was equipped with two LiDARs (Velodyne VLP-16) and an IMU, as shown in Figure 9. The LiDARs provided point cloud data with a 10 Hz sampling frequency and their maximum detection range is 100 m.

**Figure 10:**
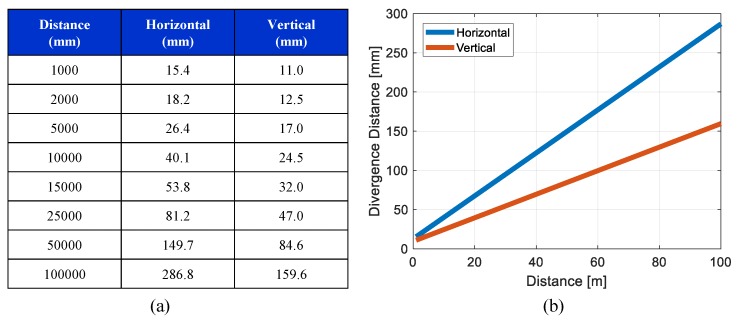
LiDAR beam divergence model: (**a**) divergence characteristics from VLP-16 specification and (**b**) divergence length over detection distance.

LiDAR lasers beams have beam divergence, which means the beam cross section is increased over time. We designed a beam divergence model based on the specification of the LiDAR sensors, as shown in Figure 10. The horizontal and vertical beam divergence characteristics were different. The standard deviation σ of the distance accuracy was set to 3 cm. Because the distance accuracy can vary based on factors such as temperature and target reflectivity, the selection of the standard deviation σ for the probabilistic LiDAR model must consider the uncertainty. The horizontal field of view (FoV) was 360${}^{\circ }$, and the horizontal angular resolution was set to 0.2${}^{\circ }$. The vertical FoV was 30${}^{\circ }$, and the vertical resolution was 2${}^{\circ }$. Because the LiDAR controls the laser emitting angle, it was assumed that the angular uncertainties (vertical and horizontal) were negligible.

Dead reckoning was implemented using the IMU to estimate the LiDAR pose for each time step. For the experiments, raw IMU data (acceleration and gyro) from the RT3002 sensor were used without real-time kinematic (RTK) GNSS correction. The specifications of the MEMS IMU are listed in Table 1. Although the IMU was not sufficiently accurate to estimate the long-term pose of the LiDAR sensor, it can provide stable performance in short windows of approximately 50 or less (five seconds or less). In addition, the evidential integration algorithm was able to account for the inaccuracy of the IMU-based pose estimation by tuning the registration confidence $\lambda_{reg}$. By considering the installed MEMS IMU, we set $\lambda_{reg}$ to 0.9.

The synchronization between the LiDAR, IMU, and point motion classification algorithm was measured by a pulse per second (PPS) signal from an RT3002. The LiDARs and IMU were precisely calibrated to be located in the same coordinate system.

### 6.2. Segmentation Performance Evaluation through Comparative Analysis

**Figure 11:**
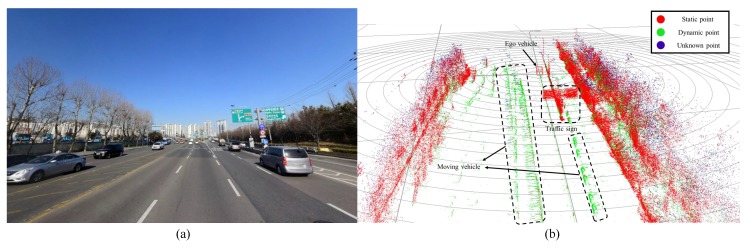
(**a**) Test site with moving cars and stationary road structures and (**b**) classification results of point-wise LiDAR motion segmentation.

To evaluate the performance of the point-wise motion segmentation, experiments were conducted under various scenarios (e.g., cities and highways). The total length of the experiment road is more than two kilometers. Figure 11a shows single scene of the experimental condition, where moving cars and stationary road structures were mixed. The result of segmentation through the proposed algorithm is shown in Figure 11b. The RGB value for each point is set using the proposed motion belief algorithm. The red values represent static state belief, the green values represent dynamic state belief, and the blue values represent unknown state belief. Therefore, the objects that have a high probability of stopping will appear red, moving objects will appear green, and unsegmented objects will appear blue. As shown in Figure 11b, traffic signs and roadside trees are segmented as red, and moving cars are classified as green.

To quantitatively analyze the segmentation performance, confusion matrices for the tracking-based classification algorithm and the proposed algorithm with various time window configurations were created, as shown in Table 2. Using the occupancy grid map, static point segmentation is possible, but dynamic point classification is not possible. Therefore, the performance of the segmentation algorithm based on the occupancy grid map is not included in the confusion matrix. For the segmentation using the proposed algorithm, the point motion is classified as static or dynamic when the belief of static and dynamic is over 0.8, respectively. The true class of points used as a reference for evaluation was classified manually. Although public data is more appropriate for comparing performance with other algorithms, there is no public data labeled by point-wise motion to verify the performance of real-time motion classification. The object tracking-based point motion segmentation algorithm segments a point as dynamic when it is located inside the bounding box of the track above a certain speed, and the remaining points not included in the moving track are segmented as static. However, the performance of point-wise motion segmentation is not superior due to incorrect bounding boxes and inaccurate speed estimation by the tracker. The segmentation accuracy of the proposed algorithm is better than that of the tracking-based segmentation algorithm when the time window *W* is 20, 30, 50, and 100, as shown in Table 2. The segmentation performance for a time window of 50 is better than that of 100 because the drift error of pose estimation affects the segmentation performance.

### 6.3. Real-Time Performance Evaluation

**Figure 12:**
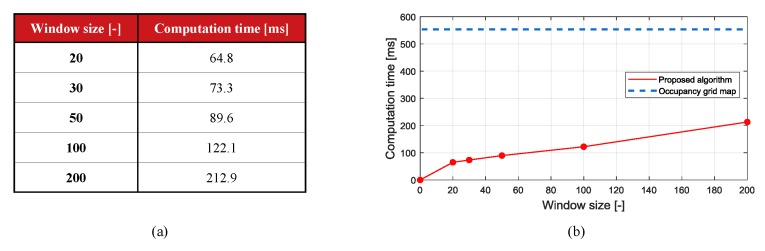
(**a**) table and (**b**) plot of Computation time for the occupancy grid map and the proposed algorithm.

The algorithm was verified in an RTMaps environment with a QuadCore Intel Core i5-3570K, 3600 MHz (36×100) CPU. Hard real-time performance could not be fully evaluated because it is not an embedded environment, but it can be optimized later by checking the soft real-time performance in the RTMaps environment. To evaluate the real-time performance of the algorithm, the occupancy grid map-based segmentation algorithm was compared with the proposed algorithm. As shown in Figure 12, the algorithm based on occupancy grid maps took a long time because all cells in the area the LiDAR beam passed were constantly updated. The larger the window size of the proposed algorithm, the more computation is required. The most appropriate time window setting for the proposed algorithm is 50, as illustrated by the confusion matrix, and its computation time is below the sampling period of the Velodyne LiDAR (100 milliseconds).

## 7. Conclusions

This paper proposed a segmentation algorithm to rapidly classify the motion states of a LiDAR point cloud in real-time. The motion segmentation algorithm requires inputs of point clouds and 3D pose (position and direction) of the LiDAR sensor. The point-wise motion segmentation is performed based on the laser beam characteristics and the 3D pose correlation between consecutive LiDAR points. A combination of probability and evidence theory is used to accurately and reliably segment the motion state of points into dynamic, static, and unknown.

(1) The point motion segmentation algorithm considers the characteristics of the LiDAR laser beam, such as multi-echo, beam divergence, and horizontal and vertical resolution. Therefore, the point motions are segmented more accurately and reliably than by conventional algorithms (e.g., occupancy grid mapping and tracking-based segmentation algorithm).

(2) To update the motion state of each LiDAR point, a combination of probability theory and evidence theory is applied to point motion modeling to accurately reflect the LiDAR characteristics. Probability theory is used to model the likelihood fields of LiDAR point clouds, taking into account the uncertainty of LiDAR measurements. Evidence theory is used to incorporate multipoint motion probabilities, taking into account pose uncertainty and the unknown state.

(3) The proposed point motion segmentation algorithm was evaluated experimentally. The segmentation accuracy was 86% for a time window of W=50. This is better than the accuracy of the tracking-based algorithm (73%). Because the proposed algorithm can handle one LiDAR point clouds in a one-step process, when operating under 100 ms, it is suitable for real-time applications in automated and intelligent vehicle systems.

The performance of the algorithm is related to the positioning algorithm that estimates the pose of the LiDAR sensor. In future research, we plan to analyze the quantitative relationship between the LiDAR sensor positioning and the proposed algorithm performance, and to study the SLAM algorithm that classifies the point motion and simultaneously estimates the pose of the LiDAR sensor.

## Figures and Tables

**Table 1:**
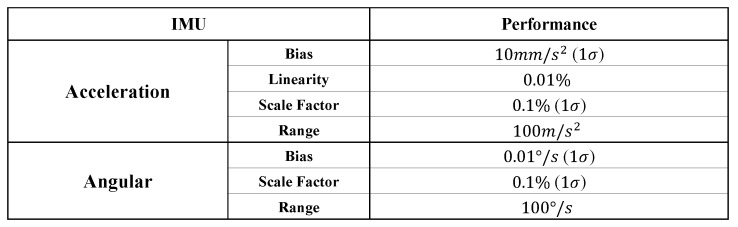
Specification of IMU sensor. An IMU was used inside the GNSS/INS (RT3002) to estimate the pose of the LiDAR sensor.

**Table 2:**
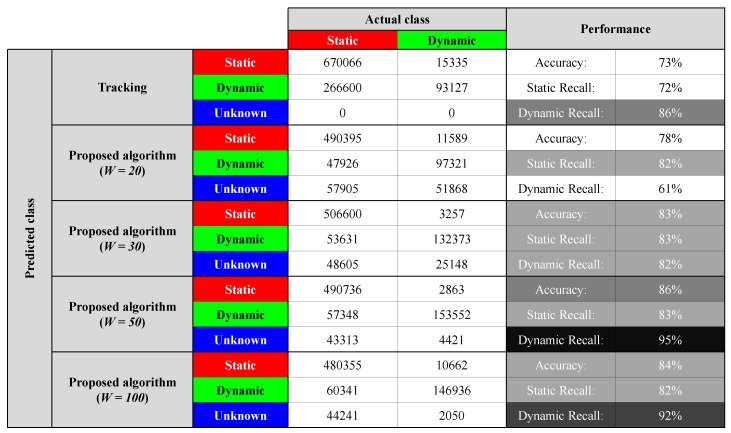
Confusion matrices of the tracking-based algorithm and the proposed algorithm with various time window configurations.
